# Supplementary material for: The Impact of Different Types of Exercise on Executive Functions in Overweight/Obese Individuals: A Systematic Review and Network Meta-Analysis
Source: Behav Sci (Basel). 2024 Dec 19;14(12):1227. doi: 10.3390/bs14121227 (PMC11673064; doi:10.3390/bs14121227)
Supplement: Supplementary file 1 [file behavsci-14-01227-s001.zip › behavsci-3330595-supplementary.pdf]

**Content**	**Pages**
Table S1. Search strategy.	2-3
Table S2. Quality assessment of included studies.	4-7
Figure S2. The funnel plot graphics of EFs.	8
Figure S1. Plot of Forest for all included interventions' eligible comparisons in the conducted analysis.	9

Database	Search Terms				
	Exercise	Obesity/overweight	Executive function	Randomized controlled trial	Note
Web of Science	(((((((((((TS=(exercise)) OR TS=(physical activity)) OR TS=(aerobic exercise)) OR TS=(moderate intensity continuous training)) OR TS=(resistance training)) OR TS=(resistance exercise)) OR TS=(strength training)) OR TS=(strength exercise)) OR TS=(strength exercise)) OR TS=(concurrent training)) OR TS=(high intensity interval training)) OR TS=(walking)) OR TS=(sports)	((TS=(obesity)) OR TS=(overweight)) OR TS=(obese)	((((((TS=(executive function)) OR TS=(working memory)) OR TS=(inhibitory control)) OR TS=(cognitive flexibility)) OR TS=(planning)) OR TS=(reasoning)) OR TS=(problem solving)	((TS=(randomized controlled trial)) OR TS=(randomized)) OR TS=(placebo)	2287 Review：244 Before 2000：78 No English: 30 No full text：84 Remain：1884
PubMed	(((((((((((exercise[Title/Abstract]) OR (physical activity[Title/Abstract])) OR (aerobic exercise[Title/Abstract])) OR (moderate intensity continuous training[Title/Abstract])) OR (resistance training[Title/Abstract])) OR (resistance exercise[Title/Abstract])) OR (strength training[Title/Abstract])) OR (strength exercise[Title/Abstract])) OR (concurrent training[Title/Abstract])) OR (high intensity interval training[Title/Abstract])) OR (walking[Title/Abstract])) OR (sports[Title/Abstract])	((obesity[Title/Abstract]) OR (overweight[Title/Abstract])) OR (obese[Title/Abstract])	((((((executive function[Title/Abstract]) OR (working memory[Title/Abstract])) OR (inhibitory control[Title/Abstract])) OR (cognitive flexibility[Title/Abstract])) OR (planning[Title/Abstract])) OR (reasoning[Title/Abstract])) OR (problem solving[Title/Abstract])	((randomized controlled trial[Title/Abstract]) OR (randomized[Title/Abstract])) OR (placebo[Title/Abstract])	123
SPORT Discus (SU)	exercise OR "physical activity" OR "aerobic exercise" OR "moderate intensity continuous training"OR "resistance training" OR " resistance exercise"OR "strength training" OR "strength exercise"OR "concurrent training" OR "high	obesity OR overweight OR obese	"executive function" OR "working memory" OR "inhibitory control" OR "cognitive flexibility" OR planning OR reasoning OR "problem solving"	"randomized controlled trial" OR randomized OR placebo	8

	intensity interval training” OR walking OR sports				
MEDLINE (SU)	exercise OR “physical activity” OR “aerobic exercise” OR “moderate intensity continuous training”OR “resistance training” OR “ resistance exercise”OR “strength training” OR “strength exercise”OR “concurrent training” OR “high intensity interval training” OR walking OR sports	obesity OR overweight OR obese	“executive function” OR “working memory” OR “inhibitory control” OR “cognitive flexibility” OR planning OR reasoning OR “problem solving”	“randomized controlled trial” OR randomized OR placebo	9
CINAHL (title abstract keyword)	exercise OR “physical activity” OR “aerobic exercise” OR “moderate intensity continuous training”OR “resistance training” OR “ resistance exercise”OR “strength training” OR “strength exercise”OR “concurrent training” OR “high intensity interval training” OR walking OR sports	obesity OR overweight OR obese	“executive function” OR “working memory” OR “inhibitory control” OR “cognitive flexibility” OR planning OR reasoning OR “problem solving”	“randomized controlled trial” OR randomized OR placebo	24

Table S1. Search strategy for Pubmed, Web of science, SPORTDiscus, MEDLINE and CINAHL.

	Study;country/region	Jadad				
		Randomization	Concealment of allocation	Double blinding	Withdrawals and dropouts	Total
1	Abel et al.,2023;Spain[1]	2	2	2	1	7
2	Furlano et al.,2023;Canada[2]	1	1	1	1	4
3	Domal et al.,2023;India[3]	2	2	2	1	7
4	Chou et al.,2023;China[4]	2	1	1	1	5
5	Oliveira et al.,2022;Brazil[5]	2	1	1	1	5
6	Zhang et al.,2022;China[6]	1	1	1	0	3
7	Zlibinaite et al.,2021;Lithuanian[7]	2	1	1	0	4
8	Zlibinaite et al.,2020;Lithuanian[8]	1	1	1	1	4
9	chou et al.,2020;China[9]	1	1	0	1	3
10	Zhang et al.,2020;China[10]	2	1	1	1	5
11	inoue et al.,2020[11]	1	1	1	1	4
12	Quintero Gacharná et al.2018[12]	1	0	0	1	2
13	Liu et al.,2018;China[13]	2	1	1	1	5
14	Allom et al.,2018;Australia[14]	2	2	1	1	6
15	Chen et al.,2017;China[15]	1	1	1	1	4
16	Wennberg et al.,2015;Australia[16]	2	2	2	0	6
17	chen et al.,2016;China[17]	2	1	1	1	5
18	Dao et al.,2013;Canada[18]	2	1	1	1	5
19	Davis et al.,2011;USA[19]	2	1	2	1	6
20	Smith et al.,2010;USA[20]	1	1	2	1	5

Table S2. Quality assessment of included studies.

Randomization:

0:not randomized or inappropriate method of randomization;

1: the study was described as randomized;

2:the method of randomization was described and it was appropriate.

Concealment of allocation:

0: Not describe the method of allocation concealment;

1: The study was described as using allocation concealment method;

2: The method of allocation concealment was described appropriately.

Double blinding:

0: No blind or inappropriate method of blinding;

1: The study was described as double blind;

2: The method of double blinding was described and it was appropriate.

Withdrawals and dropouts:

0: Not describe the follow-up;

1: A description of withdrawals and dropouts.

Included researches bibliography

1. Plaza-Florido, A. *et al.* Gene-exercise interaction on brain health in children with overweight/obesity: the ActiveBrains randomized controlled trial. *Journal of applied physiology (Bethesda, Md. : 1985)* **135**, 775–785 (2023).
2. Mora-Gonzalez, J. *et al.* The effects of an exercise intervention on neuroelectric activity and executive function in children with overweight/obesity: The ActiveBrains randomized controlled trial. *SCANDINAVIAN JOURNAL OF MEDICINE & SCIENCE IN SPORTS* (2023) doi:10.1111/sms.14486.
3. Furlano, J. A., Horst, B. R., Petrella, R. J., Shoemaker, J. K. & Nagamatsu, L. S. Changes in Cognition and Brain Function After 26 Weeks of Progressive Resistance Training in Older Adults at Risk for Diabetes: A Pilot Randomized Controlled Trial. *CANADIAN JOURNAL OF DIABETES* **47**, 250–256 (2023).
4. Domal, S. V., Chandrasekaran, B. & Palanisamy, H. P. Influence of smartphone-based physical activity intervention on executive functions and cardiometabolic disease risk in obese young adults: a pilot randomised controlled trial. *JOURNAL OF DIABETES AND METABOLIC DISORDERS* (2023) doi:10.1007/s40200-023-01182-9.
5. Chou, C.-C. *et al.* Cognitively engaging movement games improve interference control and academic performance in overweight children: A randomized control trial. *SCANDINAVIAN JOURNAL OF MEDICINE & SCIENCE IN SPORTS* **33**, 521–534 (2023).
6. Ortega, F. B. *et al.* Effects of an Exercise Program on Brain Health Outcomes for Children With Overweight or Obesity The ActiveBrains Randomized Clinical Trial. *JAMA NETWORK OPEN* **5**, (2022).
7. Oliveira, G. T. A. *et al.* Effects of 12 weeks of high-intensity interval, moderate-intensity continuous and self-selected intensity exercise training protocols on cognitive inhibitory control in overweight/obese adults: A randomized trial. *EUROPEAN JOURNAL OF SPORT SCIENCE* **22**, 1724–1733 (2022).
8. Zhang, L. *et al.* Effects of Acute High-Intensity Interval Exercise and High-Intensity Continuous Exercise on Inhibitory Function of Overweight and Obese Children. *Int. J. Environ. Res. Public Health* **19**, 10401 (2022).

9. Zlibinaite, L., Skurvydas, A., Kilikeviciene, S. & Solianik, R. Two Months of Using Global Recommendations for Physical Activity Had No Impact on Cognitive or Motor Functions in Overweight and Obese Middle-Aged Women. *JOURNAL OF PHYSICAL ACTIVITY & HEALTH* **18**, 52–60 (2021).
10. Žlibinaitė, L., Solianik, R., Vizbaraitė, D., Mickevičienė, D. & Skurvydas, A. The Effect of Combined Aerobic Exercise and Calorie Restriction on Mood, Cognition, and Motor Behavior in Overweight and Obese Women. Journal of Physical Activity and Health 17, 204–210 (2020).
11. Zhang, L. et al. Acute coordinative exercise ameliorates general and food-cue related cognitive function in obese adolescents. J Sports Sci 38, 953–960 (2020).
12. Inoue, D. S. et al. Acute increases in brain-derived neurotrophic factor following high or moderate-intensity exercise is accompanied with better cognition performance in obese adults. SCIENTIFIC REPORTS 10, (2020).
13. Liu, J.-H. et al. A randomized controlled trial of coordination exercise on cognitive function in obese adolescents. PSYCHOLOGY OF SPORT AND EXERCISE 34, 29–38 (2018).
14. Allom, V., Mullan, B., Smith, E., Hay, P. & Raman, J. Breaking bad habits by improving executive function in individuals with obesity. BMC PUBLIC HEALTH 18, (2018).
15. Chen, F.-T., Chen, S.-R., Chu, I.-H., Liu, J.-H. & Chang, Y.-K. Multicomponent Exercise Intervention and Metacognition in Obese Preadolescents: A Randomized Controlled Study. JOURNAL OF SPORT & EXERCISE PSYCHOLOGY 39, 302–312 (2017).
16. Wennberg, P. et al. Acute effects of breaking up prolonged sitting on fatigue and cognition: a pilot study. BMJ OPEN 6, (2016).
17. Chen, L.-J., Fox, K. R., Ku, P.-W. & Chang, Y.-W. Effects of Aquatic Exercise on Sleep in Older Adults with Mild Sleep Impairment: a Randomized Controlled Trial. Int J Behav Med 23, 501–506 (2016).
18. Dao, E. et al. Change in Body Fat Mass Is Independently Associated with Executive Functions in Older Women: A Secondary Analysis of a 12-Month Randomized Controlled Trial. PLoS ONE 8, e52831 (2013).
19. Davis, C. L. et al. Exercise improves executive function and achievement and alters brain activation in overweight children: A randomized, controlled trial. Health Psychology 30, 91–

98 (2011).

20. Smith, P. J. et al. Effects of the Dietary Approaches to Stop Hypertension Diet, Exercise, and Caloric Restriction on Neurocognition in Overweight Adults With High Blood Pressure. HYPERTENSION 55, 1331-U85 (2010).

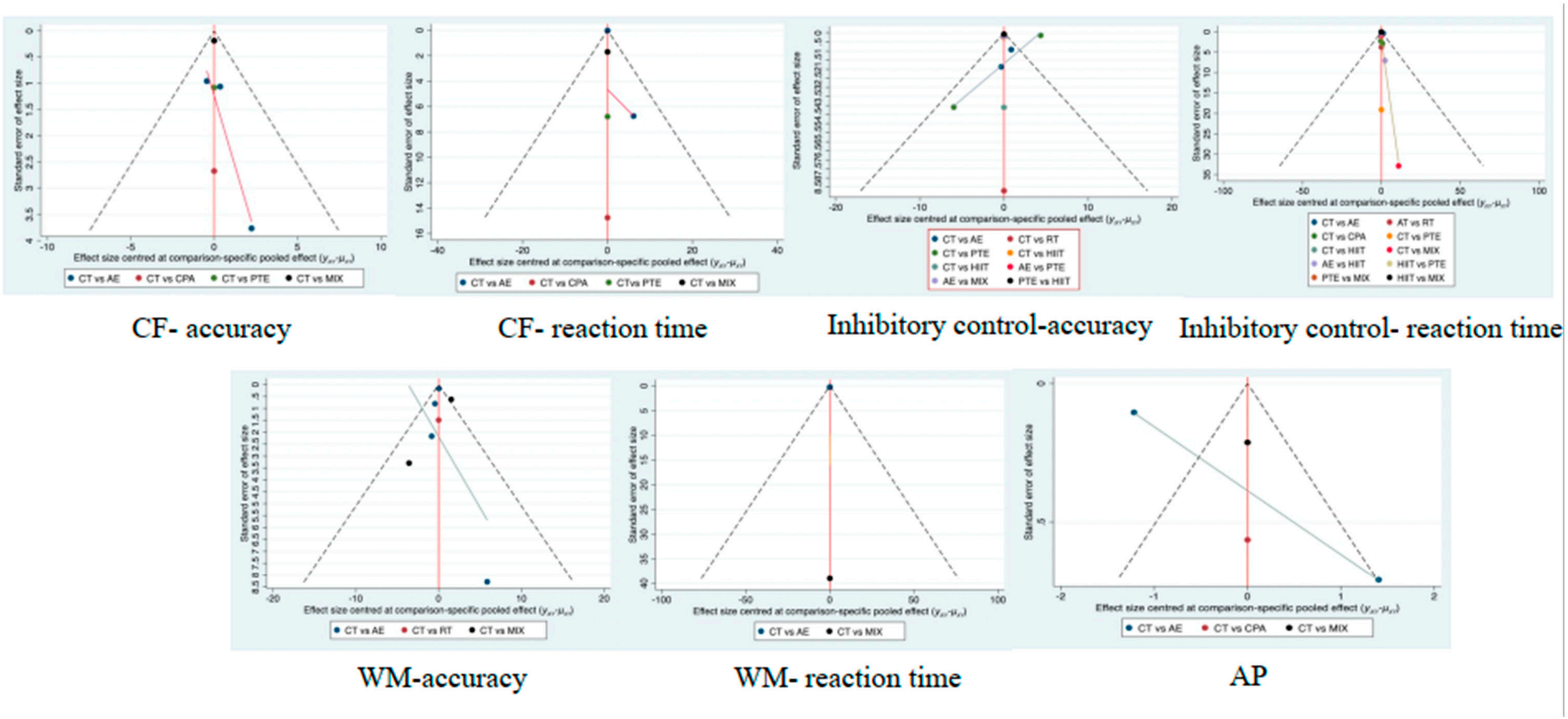

Figure S1. The funnel plot graphics of EFs. CF cognitive flexibility, WM working memory, AP academic performance.

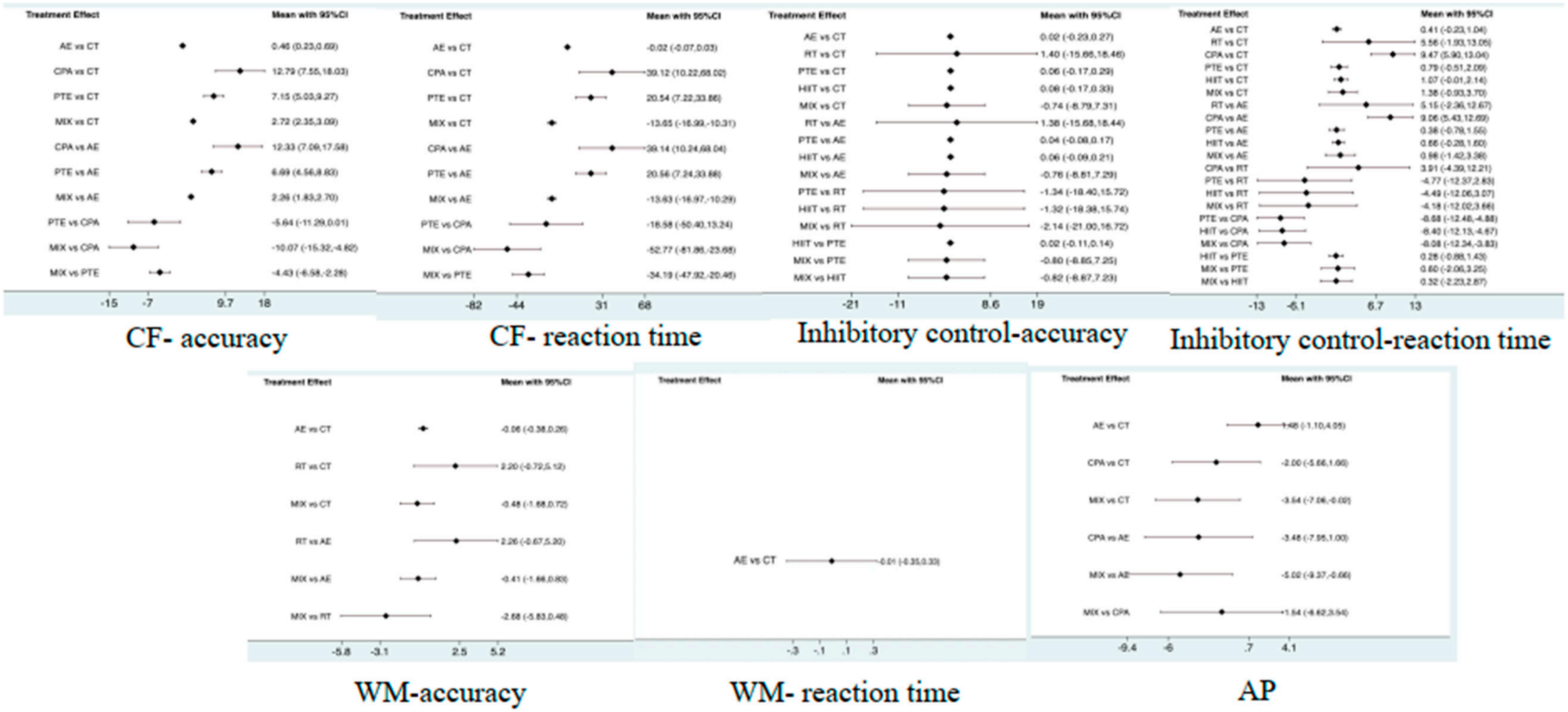

Figure S2. Plot of Forest for all included interventions' eligible comparisons in the conducted analysis. CF cognitive flexibility, WM working memory, AP academic performance.
